# Konjac glucomannan enhances 5-FU-induced cytotoxicity of hepatocellular carcinoma cells via TLR4/PERK/CHOP signaling to induce endoplasmic reticulum stress

**DOI:** 10.32604/or.2022.027584

**Published:** 2023-01-31

**Authors:** YONGKANG SHI, JUN MA, KE CHEN, BIN CHEN

**Affiliations:** Department of General Surgery, Cancer Center, Division of Gastrointestinal and Pancreatic Surgery, Zhejiang Provincial People’s Hospital, Affiliated People’s Hospital, Hangzhou Medical College, Hangzhou, 310011, China

**Keywords:** Konjac glucomannan, 5-FU, Hepatocellular carcinoma, Drug resistance

## Abstract

5-Fluorouracil (5-FU) is a commonly used chemotherapeutic agent for various cancers. However, the drug resistance developed by tumor cells hinders the therapeutic effect. Konjac glucomannan (KGM) is indicated to sensitize 5-FU-resistant hepatocellular carcinoma (HCC) cells to 5-FU. In our study, we found that KGM or 5-FU treatment alone did not affect the malignant cell behaviors and endoplasmic reticulum (ER) stress of 5-FU-resistant HCC cells or HepG2/5-FU and Bel-7402/5-FU cells, while cotreatment with KGM and 5-FU significantly facilitated HCC cell apoptosis and ER stress and suppressed cell proliferation potential and migration abilities. Moreover, we explored the underlying mechanism by which KGM induces 5-FU cytotoxicity in HCC cells. We found that Toll-like receptor 4 (TLR4) was downregulated in KGM- and 5-FU-treated HCC cells. TLR4 overexpression reversed the KGM and 5-FU cotreatment-induced inhibition of the malignant behaviors of 5-FU-resistant HCC cells. Furthermore, KGM enhanced 5-FU-induced ER stress by inhibiting TLR4 to activate PERK/ATF4/CHOP signaling. Xenograft mouse models were established using HepG2/5-FU cells, and KGM was demonstrated to reverse 5-FU resistance in HCC tumors *in vivo* by suppressing TLR4 to enhance ER stress and activate PERK/ATF4/CHOP signaling. In conclusion, KGM combined with 5-FU treatment significantly promoted apoptosis and reduced cell proliferation, migration and ER stress in 5-FU-resistant HCC cells compared with KGM or 5-FU treatment alone by downregulating TLR4 to activate PERK/ATF4/CHOP signaling.

## Introduction

Hepatocellular carcinoma (HCC) comprises 75%–85% of primary liver cancer cases and is one of the leading causes of cancer death globally [1]. HCC is affected by chronic HBV or HCV infections, foods contaminated by aflatoxin, alcohol consumption, obesity, type 2 diabetes, and cigarette smoking [2–4]. Despite improved treatment, the prognosis is still dismal because of the high recurrence risk [5]. Moreover, the efficacy of chemotherapy is often limited by multidrug resistance (MDR) [6]. Thus, the exploration of potent chemotherapeutic agents provides novel options for HCC treatment.

5-Fluorouracil (5-FU) is an antimetabolite drug commonly used in cancer therapy, including in colorectal cancer [7], gastric cancer [8] and HCC [9]. Over the past decades, combination regimens have been explored to overcome the resistance of cancer cells to 5-FU and improve antitumor activity. For example, the mTORC1/mTORC2 inhibitor AZD8055 synergistically improves the effects of 5-FU on HCC progression by reversing 5-FU-stimulated P-gp upregulation [10]. H1, as a derivative of tetrandrine, enhances 5-FU efficiency by inhibiting STAT3/MCL-1 and inducing PUMA in HCC [11]. Sorafenib at a noncytotoxic concentration inhibits 5-FU-stimulated Nrf2 expression to rescue 5-FU resistance [12].

Konjac glucomannan (KGM) is a water-soluble dietary fiber derived from *Amorphophallus konjac* K. Koch, a plant in traditional Chinese medicine (TCM). Previous studies have reported the application of KGM for the treatment of obesity with good safety [13]. The MDR acquired by tumor cells hinders the efficacy of chemotherapy. KGM enhances the chemosensitivity of 5-FU-resistant HCC cells by inactivating AKT signaling [14]. Additionally, KGM inhibits HCC cell viability and proliferation potential and is suggested to be an anticancer agent for liver cancer [15]. A recent study has revealed that KGM as dietary supplement or adjuvant suppresses breast cancer tumor growth and decreases regulatory T cell response by downregulating TGF-β and Foxp3 in the tumor microenvironment [16]. A systematic study also indicated that KGM can impair cell survival and induce autophagy in tumor cells, showing great potential in antitumor therapy [17]. However, the effect of KGM on overcoming 5-FU resistance has not been fully investigated, and more potential underlying mechanisms remain to be explored.

Endoplasmic reticulum (ER) stress is triggered when tumor cells are exposed to intrinsic and external factors and is crucially involved in cancer progression and resistance to treatment [18]. Increasing evidence has revealed that ER stress is associated with 5-FU resistance in various cancers. For example, ER stress confers 5-FU resistance in breast cancer cells by regulating the GRP78/OCT4/lncRNA MIAT/AKT pathway [19]. ER stress is reported to induce 5-FU resistance in colon cancer, and the silencing of GRP78, ATF6, ERK, or AKT is indicated to increase the sensitivity of colon cancer cells to 5-FU [20]. PGC-1α improves the survival of the 5-FU-resistant CRC cells by modulating mitochondrial function and ER stress under 5-FU exposure [21]. Toll like receptor 4 (TLR4) is highly expressed in HCC cells and promotes HCC cell growth and tumorigenesis via the activation of ERK1/2 signaling [22]. A study has also revealed that TLR4 silencing reduces 5-FU resistance in colorectal cancer cells via ERK signaling [23]. However, the role of TLR4/PERK/CHOP signaling in the 5-FU resistance of hepatocellular carcinoma remains unclear and requires further exploration.

In the current work, we intended to reveal the effects and underlying mechanism of KGM on 5-FU-induced cytotoxicity in HCC cells. *In vivo* xenograft mouse models demonstrated that KGM reverses 5-FU resistance in HCC tumors by inhibiting TLR4 to increase ER stress and activate PERK/ATF/CHOP signaling. We hypothesized that KGM enhanced 5-FU-induced ER stress to improve the cytotoxicity of 5-FU to HCC cells. The findings of our study may provide clues to overcome MDR in HCC.

## Materials and Methods

### Cell culture and transfection

Hepatocellular carcinoma HepG2 and Bel-7402 cells were provided by the American Type Culture Collection (ATCC, Manassas, VA, USA). HCC cells were incubated in Roswell Park Memorial Institute (RPMI) 1640 medium (Sigma‒Aldrich) with 10% FBS and 1:100 penicillin/streptomycin at 37°C with 5% CO_2_. HCC cells with 5-FU resistance (HepG2/5-FU, Bel-7402/5-FU) were induced as previously described [24,25] via coincubation with gradient concentrations of 5-FU (Sigma‒Aldrich) in the cell medium. The following assays used Bel-7402/5-FU and HepG2/5-FU cells in logarithmic growth phase.

To overexpress TLR4, pcDNA3.1/TLR4 vectors were designed and synthesized by Sangon Biotech (Shanghai, China) with pcDNA3.1 empty vectors as the negative control. The 5-FU-resistant HCC cells were grown into six-well plates and transfected with pcDNA3.1/TLR4 or pcDNA3.1 empty vectors with Lipofectamine 3000.

### Xenograft mouse models

Twenty-four athymic male BALB/c nude mice were provided by Beijing Vital River Laboratory Animal Technology Co., Ltd. (Beijing, China). The mice were kept at 24°C–26°C in a 12 h light/dark cycle with free access to food and water. The procedures in the animal experiment were approved by the Ethics Committee of Zhejiang Provincial People’s Hospital. The mice were randomly separated into the control, KGM+5-Fu, and KGM+5-FU+pcDNA3.1/TLR4 groups (n = 8 per group). To establish xenograft mouse models, each mouse was subcutaneously injected with HepG2/5-FU cells (150 µl, 4 × 10^6^ cells) into the right flank. We monitored and recorded the mouse tumor volume every seven days. When the tumor volume reached 40–50 mm^3^, the mice received treatment with 2 mg/kg 5-FU and 20 mg/kg KGM via intraperitoneal injection every two days [14]. After 4 weeks, the mice were killed via cervical dislocation, and the tumor weight in each group was measured.

### Hematoxylin-eosin (HE) staining

Mouse tumor tissues were fixed with 4% paraformaldehyde, paraffin-embedded and sliced into 5-μm sections. Then the sections were dewaxed and dehydrated in xylene for 10 min three times and washed in gradient alcohol. The sections were then stained in H solution (Beyotime) for 10 min and dyed in E solution (Beyotime) for 3 min at room temperature. A microscope was used to capture the images in each group.

### qRT‒PCR

TRIzol reagent (Thermo Fisher) was used to isolate total RNA from HCC cells under the indicated treatments. A PrimeScript™ RT Reagent Kit was applied for cDNA synthesis. PCR was performed with a qRT‒PCR kit (QR0100-1KT, Sigma‒Aldrich) with an ABI 7300 Real-Time PCR system. Relative RNA levels were quantified with the 2^−ΔΔCt^ method and normalized to GAPDH. The primer sequences are as follows:

TLR4

Forward: 5′-GGACCTGAGCTTTAATCCC-3′,

Reverse: 5′-GATTTCACACCTGGATAAATCC-3′.

### Western blot

A Total Protein Extraction Kit (Beyotime) was used to extract the proteins from tumor tissues and cells. The proteins were subjected to concentration determination with a BCA assay. Then, the proteins were loaded on sodium dodecyl sulfate‒polyacrylamide gel electrophoresis gels and electrotransferred to PVDF membranes. Subsequently, the membranes were blocked with blocking buffer (Beyotime, Shanghai, China) and then incubated with the primary antibodies anti-p-PERK (PA5-40294, 1/1000, Thermo Fisher), anti-PERK (PA5-15305, 1/1000, Thermo Fisher), anti-ATF4 (PA5-27576, 1/1000, Thermo Fisher), and anti-CHOP (PA5-88116, 1/1000, Thermo Fisher) with GAPDH as the loading control. Next, after washing with PBS and culturing with the secondary antibodies, the protein bands were visualized using an enhanced chemiluminescence detection system (Beyotime) and quantified with ImageJ software.

### Cell viability

The viability of HCC cells under the indicated treatments was subjected to Cell Counting Kit-8 (CCK-8) assays using a CCK-8 cell activity detection kit (Beyotime, Shanghai, China) following the manufacturer’s instructions. HepG2/5-FU and Bel-7402/5-FU cells were inoculated into 96-well plates, and CCK-8 solution was added to the wells (10 μL/well) at 48 h and cultured for another 2 h. The optical density was measured with a microplate reader at 450 nm.

### Cell proliferation

The proliferation potential of HCC cells resistant to 5-FU was analyzed by colony formation assays. The cells were grown in six-well plates and cultured for 14 days. Then, 4% paraformaldehyde was used to fix the cell colonies for 30 min, and 0.1% crystal violet was used for cell staining for 20 min. The colony number was calculated in three randomly chosen visual fields using a light microscope.

### Flow cytometry

The apoptosis of HCC cells resistant to 5-FU was assessed using flow cytometry analysis with an Annexin-V-FITC apoptosis detection kit. Briefly, 2.0 × 10^5^ HCC 5-FU-resistant cells after the indicated treatments were resuspended in binding buffer and cultured with Annexin-V-FITC (5 μL) and propidium iodide (PI, 10 μL) for 15 min at ambient temperature. The apoptotic rate was calculated as the percentage of early stage apoptotic cells (Q3) + late stage apoptotic cells (Q2). which was quantified by flow cytometry using CellQuest Pro software.

### Transwell

The migration ability of HCC cells resistant to 5-FU was evaluated by Transwell assays. Cells under different treatments were resuspended in RPMI medium (serum free), and 200 μL of cell suspension was added to the apical transwell chambers at 1.5 × 10^4^ cells/mL. The basolateral chambers were supplemented with RPMI medium with 10% FBS. Twenty-four hours later, methanol was used to fix the migrated cells, and crystal violet was used to stain the cells for 30 min. The number of migrated cells was calculated in five randomly chosen visual fields using an inverted microscope.

### MDA and ROS determination

The MDA levels in HCC cells resistant to 5-FU and mouse tumor tissues were assessed using an MDA Assay Kit in accordance with the manufacturers’ instructions. The tumor tissues were homogenized and centrifuged at 12,000 rpm at 4°C for 15 min. The MDA content was measured using a microplate reader with the absorbance at 532 nm.

An ROS detection kit (Beyotime) was used to detect the ROS levels in 5-FU-resistant HCC cells and tumor tissues. The 2.5 mL cells (1 × 10^5^ cells/mL) were diluted with 5% DMEM and grown in 6-well plates for 12 h. The cells were cultured with 20 μM DCFH-DA for 30 min at 37°C. The cell suspension was collected and subjected to flow cytometry analysis.

### ELISA

The 8-hydroxy-2′-deoxyguanosine (8-OHdG) levels in 5-FU-resistant HCC cells were measured using ELISAs. An 8-OHDG ELISA Kit was used to detect the relative levels of 8-OHdG according to the manufacturers’ instructions.

### Statistical analysis

The results are expressed as the means ± SD. GraphPad Prism 8.0 software was used for statistical analysis. Statistical differences were analyzed with unpaired Student’s *t*-test for two-group comparisons, and one-way analysis of variance (ANOVA) with Tukey’s *post hoc* test was used for multiple group comparisons. The significance level was set at *p* < 0.05.

## Results

### KGM enhances the 5-FU-induced cytotoxicity of HCC cells

**FIGURE 1:**
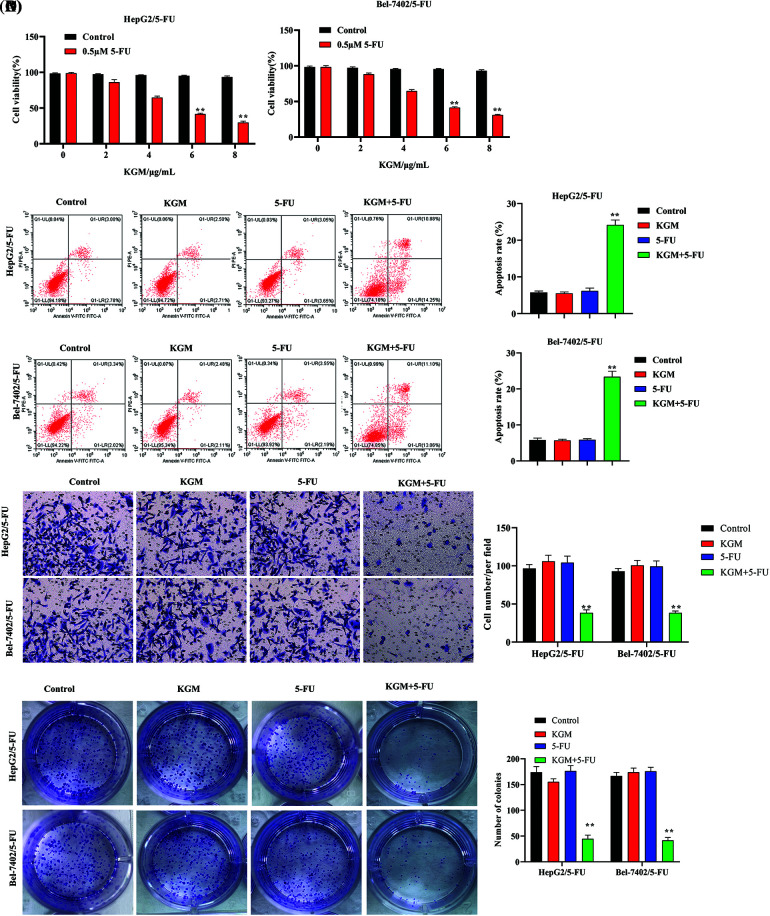
KGM enhances the 5-FU-induced cytotoxicity of HCC cells. (A) CCK-8 assays were used to measure the viability of HepG2 and Bel-7402 5-FU-resistant cells (HepG2/5-FU and Bel-7402/5-FU cells) under treatment with different doses of KGM (0, 2, 4, 6, and 8 μg/mL). (B) The apoptosis of 5-FU-resistant HCC cells in different groups was analyzed using flow cytometry. (C) Transwell assays were used to evaluate cell migration ability under the indicated treatments. (D) The cell proliferation potential was measured using colony formation assays. **p < 0.01.

The viability of HepG2 and Bel-7402 5-FU-resistant cells was measured using CCK-8 assays. KGM treatment alone showed no significant influence on the viability of HepG2 and Bel-7402 cells. However, the cell viability of HepG2/5-FU and Bel-7402/5-FU cells was significantly reduced by KGM in a concentration-dependent manner, and KGM at 6 μg/mL and 8 μg/mL did not exhibit a significant difference in viability inhibition (Fig. 1A). The apoptosis of 5-FU-resistant HCC cells was analyzed using flow cytometry, which revealed that KGM or 5-FU alone did not significantly affect the HCC cell apoptosis rate, while the combination showed a synergistic effect that significantly elevated the apoptosis rate of HCC cells (Fig. 1B). As exhibited by Transwell assays, the migration of HepG2/5-FU and Bel-7402/5-FU cells was not affected under KGM or 5-FU treatment alone, while the combined treatment of KGM and 5-FU significantly suppressed the migration of 5-FU-resistant HCC cells (Fig. 1C). Similarly, the colony formation potential of Bel-7402/5-FU and HepG2/5-FU cells showed no significant difference in the KGM or 5-FU treatment groups. However, a synergistic effect was observed, and the colony number of Bel-7402/5-FU and HepG2/5-FU cells was significantly reduced under the combined treatment of KGM and 5-FU (Fig. 1D).

### KGM enhances 5-FU-induced ER stress via PERK/ATF4/CHOP signaling

**FIGURE 2:**
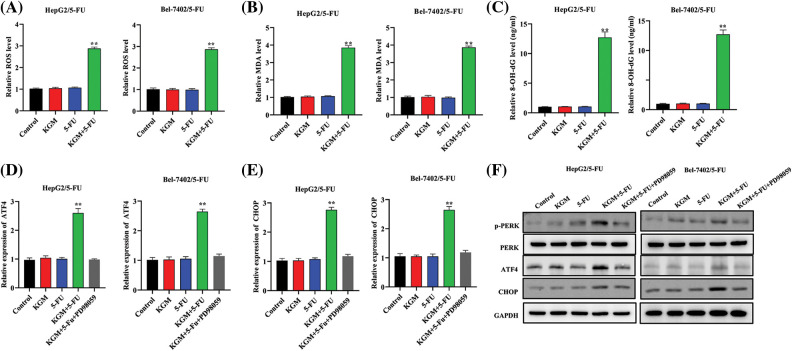
KGM enhances 5-FU-induced ER stress via PERK/ATF4/CHOP signaling. (A) HCC cell ROS levels under different treatments (0.5 μM 5-FU, 6 μg/mL KGM) were measured by flow cytometry. (B–C) Malondialdehyde (MDA) and 8-hydroxy-2′-deoxyguanosine (8-OHdG) levels in Bel-7402/5-FU and HepG2/5-FU cells under the indicated treatments. (D and E) ATF4 or CHOP mRNA levels in HCC cells resistant to 5-FU or under treatment with PD98058 (20 μM). (F) The protein expression of p-PERK, PERK, ATF4 and CHOP under different treatments in Bel-7402/5-FU and HepG2/5-FU cells. **p < 0.01.

ER stress is critically involved in cancer progression, and 5-FU is reported to induce ER stress as well as the consequent intrinsic apoptosis in HCC cells [26]. In our study, we proposed that KGM may rescue Bel-7402/5-FU and HepG2/5-FU cell resistance by enhancing 5-FU-stimulated ER stress. As shown in Fig. 2A, HCC cell ROS levels were detected, and KGM or 5-FU stimulation alone did not affect the cell ROS levels, while cotreatment with 5-FU (0.5 μM) and KGM (6 μg/mL) significantly elevated the ROS levels of 5-FU-resistant HCC cells. Malondialdehyde (MDA) levels are a product of oxidative stress, and 8-hydroxy-2′-deoxyguanosine (8-OHdG) is a biomarker for oxidative stress-induced DNA damage. Similarly, MDA and 8-OHdG were not affected by KGM or 5-FU treatment alone and showed a significant reduction when treated with both KGM and 5-FU (Figs. 2B and 2C). Then, we explored the effect of KGM or 5-FU treatment on potential signaling pathways. The qRT‒PCR results showed that KGM or 5-FU treatment alone did not affect the relative ATF4 or CHOP mRNA levels, which were demonstrated to be significantly elevated in the KGM or 5-FU cotreatment group. Additionally, treatment with PD98058 (20 μM), an inhibitor of ERK1/2, significantly reversed the increase in ATF4 or CHOP mRNA expression in 5-FU resistant HCC cells (Figs. 2D and 2E). Moreover, western blot assays further indicated that p-PERK, PERK, ATF4 and CHOP protein levels showed no significant difference in the KGM or 5-FU treatment groups compared with the control, while their cotreatment significantly increased the protein expression of p-PERK, ATF4 and CHOP in HCC cells with 5-FU resistance. Furthermore, treatment with PD98058 significantly counteracted the increase in the protein expression of p-PERK, ATF4 and CHOP in the KGM+5-FU group (Fig. 2F). Overall, these findings suggested that KGM enhances 5-FU-induced ER stress by activating PERK/ATF4/CHOP signaling.

### KGM enhances the 5-FU-induced cytotoxicity of HCC cells by inhibiting TLR4 expression

**FIGURE 3:**
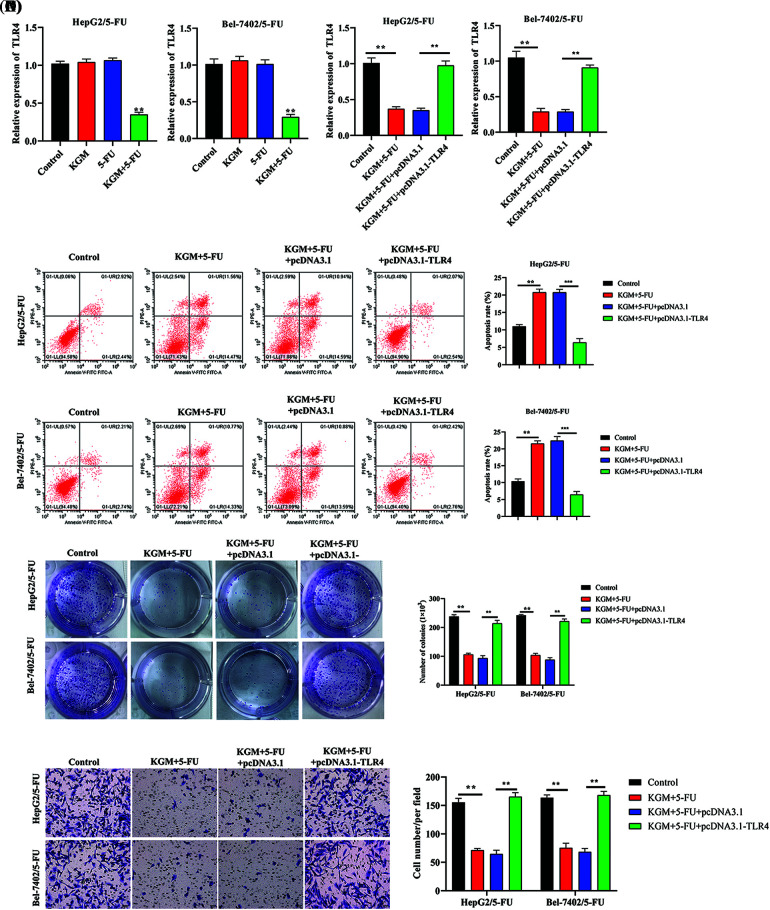
KGM enhances the 5-FU-induced cytotoxicity of HCC cells by downregulating TLR4. (A) The expression of TLR4 in 5-FU-resistant HCC cells treated with KGM or 5-FU alone or cotreated with KGM and 5-FU. (B) The overexpression efficiency of TLR4 in HCC cells with 5-FU resistance under cotreatment with KGM and 5-FU. (C) Flow cytometry was used to evaluate the apoptosis of Bel-7402/5-FU and HepG2/5-FU cells under the indicated treatments. (D) The proliferation potential of 5-FU-resistant HCC cells under the indicated treatments. (E) Transwell assays were applied to measure the migration ability of HepG2/5-FU and Bel-7402/5-FU cells with the indicated treatments. **p < 0.01, ***p < 0.001.

The expression of TLR4 was measured in HCC cells with 5-FU resistance. TLR4 levels were not affected by KGM or 5-FU treatment alone. However, after cotreatment with KGM and 5-FU, the expression of TLR4 showed a significant decrease (Fig. 3A). Then, we detected the overexpression efficiency of TLR4 in Bel-7402/5-FU and HepG2/5-FU cells. We found that the transfection of pcDNA3.1/TLR4 significantly elevated TLR4 expression in HCC cells treated with KGM and 5-FU (Fig. 3B). Moreover, the elevated apoptosis rate of Bel-7402/5-FU and HepG2/5-FU cells induced by the cotreatment of KGM and 5-FU was reversed after TLR4 overexpression (Fig. 3C). Then, we explored the effect of TLR4 on the proliferation and migration of HCC cells resistant to 5-FU. These findings demonstrated that TLR4 overexpression reversed the inhibitory effects of KGM and 5-FU cotreatment on the proliferation potential and migration ability of Bel-7402/5-FU and HepG2/5-FU cells (Figs. 3D and 3E).

### KGM enhances 5-FU-induced ER stress by suppressing TLR4 to activate PERK/ATF4/CHOP signaling

**FIGURE 4:**
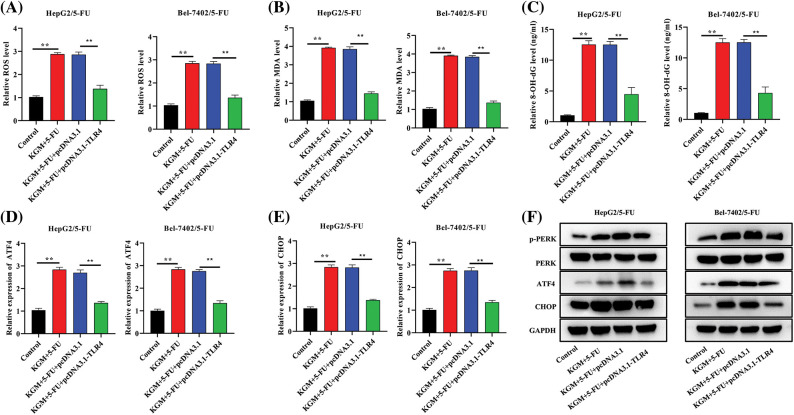
KGM enhances 5-FU-induced ER stress by suppressing TLR4 to activate PERK/ATF4/CHOP signaling. (A) ROS levels in cells under the indicated treatments. (B and C) The effect of TLR4 overexpression on MDA and 8-OHdG levels in KGM and 5-FU cotreated HepG2/5-FU and Bel-7402/5-FU cells was evaluated using an MDA Assay Kit and 8-OHdG ELISA Kit, respectively. (D and E) qRT‒PCR was used to measure ATF4 and CHOP mRNA expression in HCC cells with 5-FU resistance in the indicated groups. (F) Western blotting was used to detect the protein expression of p-PERK, PERK, ATF4 and CHOP in Bel-7402/5-FU and HepG2/5-FU cells under different treatments. **p < 0.01.

We further explored the regulatory mechanism by which KGM enhances 5-FU-induced ER stress in HCC cells resistant to 5-FU. The cotreatment of KGM and 5-FU induced an increase in ROS levels that was significantly rescued by TLR4 overexpression in Bel-7402/5-FU and HepG2/5-FU cells (Fig. 4A). Similarly, the MDA and 8-OHdG levels were increased in KGM- and 5-FU-cotreated Bel-7402/5-FU and HepG2/5-FU cells and showed a significant reduction after the transfection of TLR4 (Figs. 4B and 4C). Moreover, the KGM and 5-FU stimulation-induced increase in ATF4 and CHOP mRNA expression was reversed by TLR4 overexpression in HCC cells resistant to 5-FU (Figs. 4D and 4E). The expression of key proteins in PERK/ATF4/CHOP signaling was also analyzed, and the results indicated that p-PERK, ATF4 and CHOP protein expression was elevated after stimulation with KGM and 5-FU, while TLR4 upregulation showed a suppressive effect on these proteins (Fig. 4F). Overall, KGM enhances 5-FU-stimulated ER stress by inhibiting TLR4 expression to activate PERK/ATF4/CHOP signaling.

### KGM reversed the 5-FU resistance of HCC tumors by downregulating TLR4 in vivo

**FIGURE 5:**
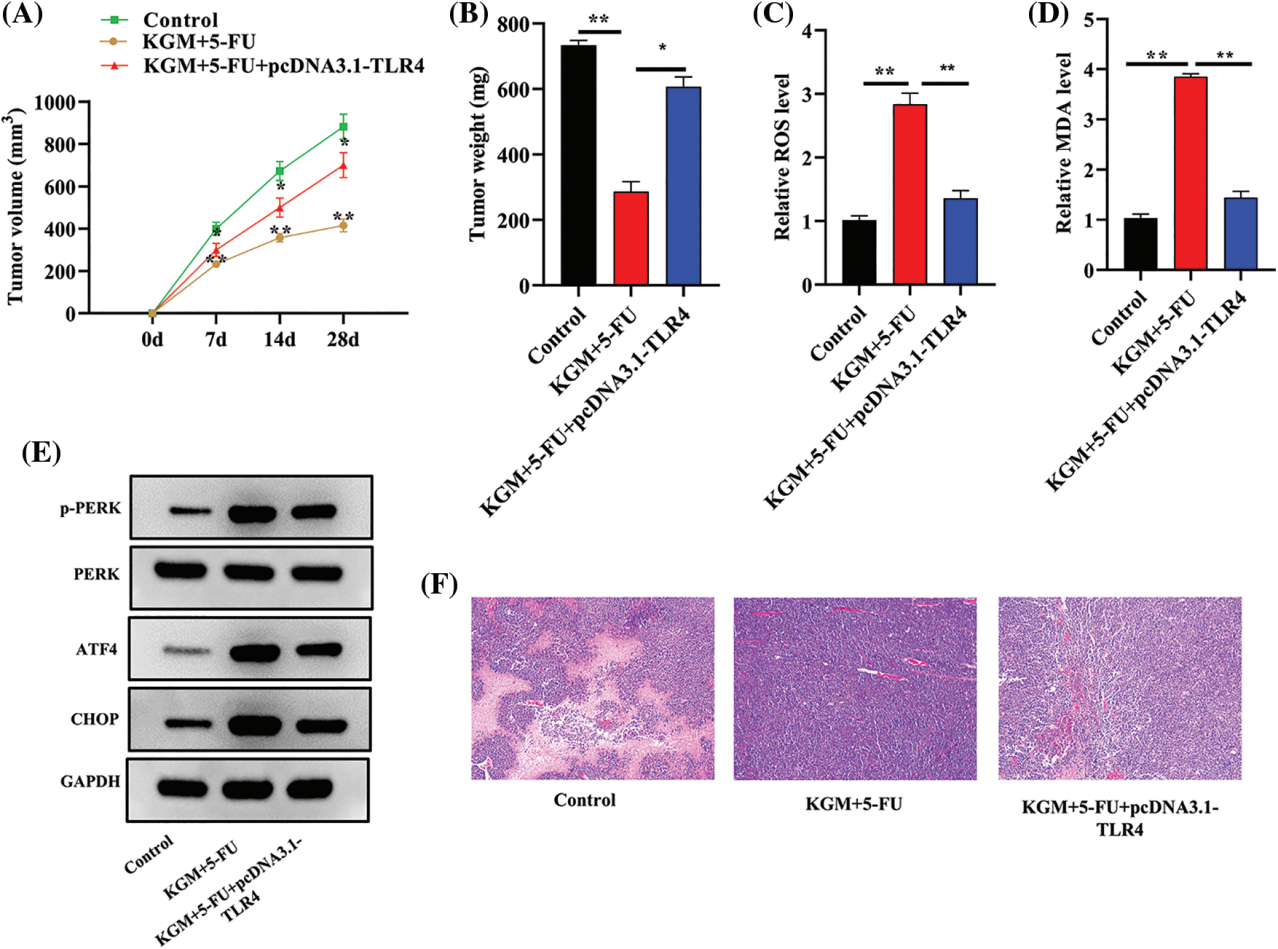
KGM reversed the 5-FU resistance of HCC tumors by downregulating TLR4 *in vivo*. (A) The tumor volume in the control, KGM+5-FU and KGM+5-FU+pcDNA3.1/TLR4 groups was measured at the indicated time points (0, 7, 14, 28 days). (B) Mouse tumor weight in the indicated groups was measured after the mice were sacrificed. (C and D) ROS and MDA levels in mouse tumor tissues. (E) p-PERK, ATF4 and CHOP protein expression in mouse tumor tissues. (F) The histological changes in mouse tumor tissues in each group were examined using HE staining. *p < 0.05, **p < 0.01.

HCC mouse models were established using HepG2/5-FU cells, and we further explored the function and mechanism of KGM in 5-FU resistance *in vivo*. As shown in Fig. 5A, the tumor volume was monitored every week and was found to be significantly smaller in the KGM+5-FU group, while TLR4 overexpression reversed the decrease in tumor volume induced by KGM and 5-FU cotreatment. Similarly, the KGM- and 5-FU-induced decrease in tumor weight was significantly reversed by TLR4 overexpression (Fig. 5B). Moreover, we also measured the ROS and MDA levels in mouse tumor tissues. The results indicated that the ROS and MDA levels both exhibited significant elevation after cotreatment with KGM and 5-FU, which was reversed by TLR4 overexpression (Figs. 5C and 5D). The protein expression of p-PERK, ATF4 and CHOP in mouse tumor tissues was evaluated, and the KGM+5-FU-induced elevation in the protein levels of p-PERK, ATF4 and CHOP was reversed after TLR4 upregulation (Fig. 5E). Moreover, the results of HE staining assays revealed that the morphological changes in mouse tumor tissues were significantly alleviated in the KGM+5-FU group, which was rescued after TLR4 overexpression (Fig. 5F).

## Discussion

In this study, the influence of KGM on 5-FU resistance in HCC progression was investigated. In HCC cells with 5-FU resistance, KGM or 5-FU treatment alone did not affect the HCC cell phenotypes, while cotreatment significantly promoted apoptosis and inhibited the proliferation and migration potential of HCC cells with 5-FU resistance. The underlying mechanisms by which KGM enhances 5-FU-induced cell cytotoxicity were further explored. KGM enhanced 5-FU-induced cell cytotoxicity and ER stress by inhibiting TLR4 to activate PERK/ATF4/CHOP signaling. Moreover, xenograft mouse models were established using HepG2/5-FU cells, and KGM was demonstrated to reverse HCC 5-FU resistance by downregulating TLR4 *in vivo*.

5-FU is commonly used in cancer chemotherapy for breast cancer, colorectal cancers and HCC [12,27]. However, drug resistance remains an obstacle in the process of chemotherapy. Previous studies have developed ways to sensitize 5-FU-resistant tumor cells to 5-FU [12,28–30], and KGM is indicated to enhance the sensitivity of HCC cells to 5-FU by facilitating apoptosis and hindering the proliferation potential by inactivating the AKT pathway and upregulating p53 levels [14]. In contrast to a previous study, we also found that KGM enhanced 5-FU-induced cell cytotoxicity by inhibiting cell growth and migration and promoting cell apoptosis. The underlying mechanism by which KGM exerts its function was further explored. KGM and 5-FU cotreatment was revealed to enhance 5-FU-induced ER stress in 5-FU-resistant HCC cells.

Moreover, we also found that TLR4 played a significant role in reversing 5-FU resistance in HCC cells. KGM was demonstrated to enhance 5-FU-induced cell cytotoxicity and ER stress by inhibiting TLR4. Numerous studies have reported the role of TLR4 as an oncogene in various cancers [31], including breast cancer [32], colon cancer [33] and HCC [34,35]. Moreover, a previous study indicated that miR-145 upregulation sensitizes HCC cells to 5-FU and enhances the 5-FU-induced inhibition of HCC tumor growth by targeting TLR4 [36]. Similarly, in our study, we found that TLR4 expression was not significantly affected by KGM or 5-FU treatment alone in 5-FU-resistant HCC cells, while the combined treatment of KGM and 5-FU exhibited significant inhibition of TLR4 expression, which was reversed by transfection of TLR4 overexpression vectors. KGM sensitized 5-FU-resistant HCC cells to 5-FU by inhibiting TLR4 expression. TLR4 overexpression reversed the cotreatment of KGM and 5-FU-induced inhibition of proliferation and migration potential and the enhancement of cell apoptosis in 5-FU-resistant HCC cells. Moreover, the ER stress enhanced by KGM and 5-FU cotreatment was reversed by TLR4 overexpression *in vitro*. *In vivo* assays revealed that mouse tumor growth and ER stress inhibited by KGM and 5-FU cotreatment were rescued after TLR4 upregulation.

ER stress is commonly observed in cancer progression due to the hypoxic and oxidative tumor environment, and continued ER stress may lead to apoptosis [18,37,38]. ER stress inhibits cancer progression via PERK/ATF4/CHOP signaling. In response to ER stress, PERK forms oligomers via autophosphorylation, and sustained ER stress activates its downstream transcription factor, ATF4, which can promote transcription and elevate CHOP expression [39,40]. ROS also play an important role in the unfolded protein response activated by ER stress [41], and ER stress-mediated oxidative stress is critically involved in cancer progression [42]. In this study, TLR4 was shown to reverse KGM and 5-FU cotreatment-induced ER stress. The expression of p-PERK, ATF4 and CHOP was significantly increased after KGM and 5-FU treatment, while TLR4 overexpression reversed the KGM+5-FU-induced activation of PERK/ATF4/CHOP signaling. *In vivo* assays also revealed that KGM sensitized HCC tumors to 5-FU by inhibiting TLR4 to reduce ROS and MDA levels and activate the PERK/ATF4/CHOP pathway.

In conclusion, KGM combined with 5-FU significantly promoted cell apoptosis, and inhibited cell proliferation, migration and ER stress compared KGM or 5-FU treatment alone by inhibiting TLR4 to activate PERK/ATF4/CHOP signaling. HCC mouse models were established, and KGM reversed 5-FU resistance by downregulating TLR4 in HCC *in vivo*.
